# P-1958. Efficacy and Safety of Rezafungin, A Long-Acting Echinocandin

**DOI:** 10.1093/ofid/ofaf695.2125

**Published:** 2026-01-11

**Authors:** Richard L Hengel, Brian S Metzger, Erika M Young, Kent J Stock, Joseph F John, Kimberly A Couch, Christina J Weeks, Lucinda J Van Anglen

**Affiliations:** Atlanta ID Group, Atlanta, GA; Austin Infectious Disease Consultants, Austin, Texas; Infectious Disease Specialists, Valparaiso, Indiana; Low Country Infectious Disease, Charleston, South Carolina; Low Country Infectious Diseases, PA, Charleston, SC; Healix Infusion Therapy, LLC, Sugar Land, Texas; Healix Infusion Therapy, LLC, Sugar Land, Texas; Healix Infusion Therapy, LLC, Sugar Land, Texas

## Abstract

**Background:**

Rezafungin (RZF), a novel long-acting intravenous echinocandin, was approved by the FDA in March 2023 for adult patients (pts) with candidemia and invasive candidiasis and safety data for up to 4 weekly doses. The once weekly dosing regimen is advantageous in outpatient settings, eliminating the requirement for central venous catheters and daily medication administration.

**Table 1.**
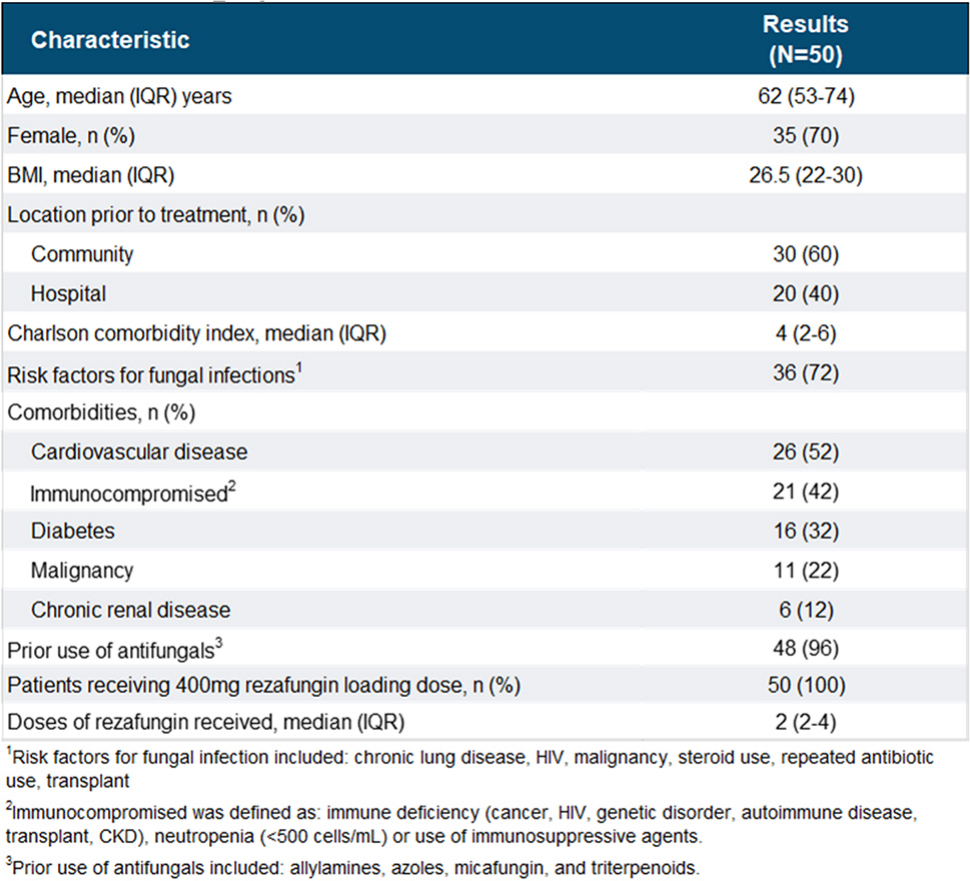
Demographics and Baseline Characteristics

**Figure 1.**
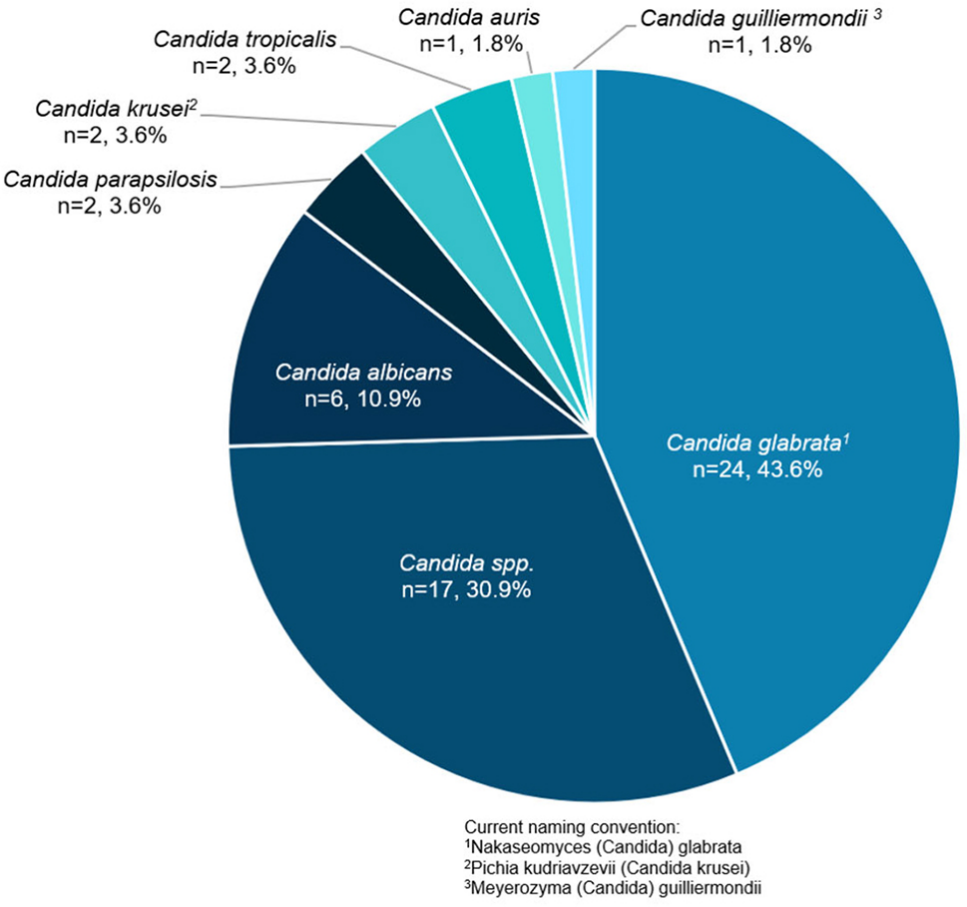
Pathogens in RZF Treatment Courses

**Methods:**

Rezafungin (RZF), a novel long-acting intravenous echinocandin, was approved by the FDA in March 2023 for adult patients (pts) with candidemia and invasive candidiasis and safety data for up to 4 weekly doses. The once weekly dosing regimen is advantageous in outpatient settings, eliminating the requirement for central venous catheters and daily medication administration.

**Figure 2.**
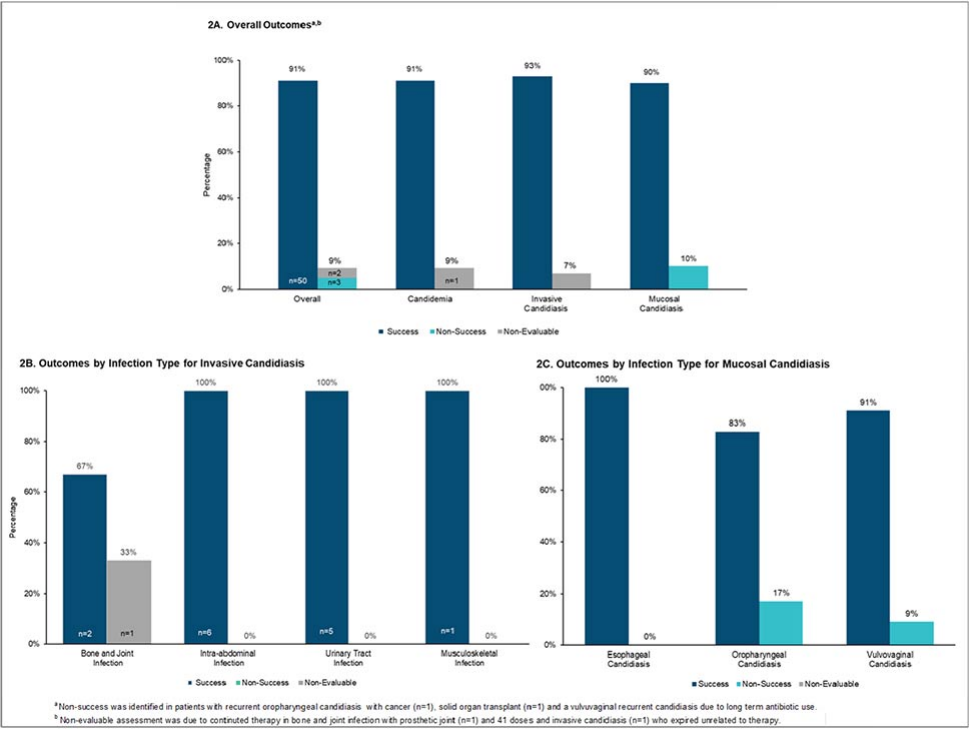
Clinical Outcomes in RZF Treatment Courses

**Results:**

50 pts received 55 treatment courses from 9/2023 to 3/2025, with characteristics in Table 1. Median age was 62 years (IQR 53-74). 20 pts (40%) received RZF following hospitalization. Median Charlson Comorbidity Index was 4 (IQR 2-6). 36 pts (72%) had risk factors for fungal infections and 48 (96%) had prior anti-fungal therapy, including all those with mucosal candidiasis. Initial dose was 400 mg and the median number of weekly doses received was 2 (IQR 2-4). RZF was used to treat candidemia (n=11), invasive candidiasis (n=15), or mucosal candidiasis (n=29). Pathogens are noted in Figure 1. *Candida glabrata* was the predominant organism. Out of 55 treatment courses, clinical success overall was achieved in 50 patient treatment courses (91%), with non-success in 3 (5%) and 2 (4%) non-evaluable. Outcomes are displayed in Figure 2, including those by diagnostic group. Non-serious adverse events occurred in 4 (7%) treatments, none requiring discontinuation.

**Conclusion:**

RZF was a safe, tolerable, and effective treatment for candidemia and candidiasis in this treatment experienced comorbid population. This real-world data establishes the successful use of a long-acting weekly echinocandin in the outpatient treatment of patients with fungal infections.

**Disclosures:**

Richard L. Hengel, MD, FIDSA, Gilead: Grant/Research Support Brian S. Metzger, MD, MPH, Abbvie: Advisor/Consultant|Cumberland Pharmaceuticals: Advisor/Consultant|Ferring Pharmaceuticals: Advisor/Consultant Lucinda J. Van Anglen, PharmD, FIDSA, Cumberland Pharmaceuticals: Grant/Research Support|Ferring Pharmaceuticals: Grant/Research Support|Melinta Therapeutics: Grant/Research Support|Novartis: Grant/Research Support|Takeda Pharmaceuticals: Grant/Research Support

